# Pectin methylesterase activities in reproductive tissues of maize plants with different haplotypes of the *Ga1* and *Ga2* cross incompatibility systems

**DOI:** 10.1007/s00497-024-00502-0

**Published:** 2024-05-03

**Authors:** Amruta R. Bapat, M. Paul Scott

**Affiliations:** 1 Interdepartmental Genetics and Genomics Program, Iowa State University, Ames, IA 50011 USA; 2 Department of Agronomy, Iowa State University, Ames, IA 50011 USA; 3 USDA Agricultural Research Service, Corn Insects and Crop Genetics Research Unit, 716 Farmhouse Lane, Ames, IA 50011 USA

**Keywords:** Cross-incompatibility, Enzyme activity, Pectin methylesterase, Pollen tube growth

## Abstract

**Key message:**

Total PME activity in reproductive tissues was related to haplotypes at maize cross incompatibility loci, suggesting that these loci function by controlling PME activity.

**Abstract:**

In maize, the pollination outcome depends on the haplotypes of the interacting male gametophyte (germinated pollen) and female sporophyte (silk) at several cross-incompatibility loci. Functional alleles (-*S* haplotypes) of the cross-incompatibility loci *Ga1* and *Ga2,* both encode two pectin methylesterases (PMEs), one that is expressed in silk and the other in pollen. We examined total PME activity in reproductive tissues containing functional and null haplotypes at the *Ga1* or *Ga2* loci. In pollinated silks, there was a correlation between total PME activity and the *-S* haplotype pollen in both *Ga1* and *Ga2* systems. We did not detect a significant relationship between PME activity and pollination outcome of either system. We re-examined previously reported active site amino acid substitutions in PMEs encoded by cross incompatibility loci. We observed that different active site substitutions are present in the pollen and silk PMEs of cross incompatibility loci and these differences are conserved across *Ga1*, *Ga2* and *Tcb-1*. This work establishes a relationship between total PME activity and the haplotypes of the *Ga1* locus in pollinated silks.

**Supplementary Information:**

The online version contains supplementary material available at 10.1007/s00497-024-00502-0.

## Introduction

Maize is a monoecious plant and usually is readily self- or cross-pollinated. Three cross incompatibility loci called *Gametophyte factor1* (*Ga1*), *Gametophyte factor2* (*Ga2*), and the *Teosinte crossing barrier1* (*Tcb1*) have been identified in maize and teosinte and are now well characterized at the molecular level (Chen et al. 2022; Lu et al. 2019; Moran Lauter et al. 2017; Zhang et al. 2018, 2023a, 2023b; Wang et al. 2022). Each of these loci have functional haplotypes that prevent fertilization by null haplotypes. The combination of haplotypes involved in a cross determines the outcome of the cross. Pollinations are categorized as compatible or incompatible based on whether fertilization is successful or prevented.

Compatible pollinations involve normal pollen germination on the stigma and the development of a pollen tube that successfully enters the transmitting tract of the stigma. Pollen tube growth is then sustained over several hours through the transmitting tract, ultimately reaching the female gametophyte where fertilization occurs. In the case of incompatible pollinations, the pollen lands on the stigma and germinates successfully. The pollen tube grows normally through the transmitting tract initially, but begins growing abnormally, often leaving the transmitting tract, and eventually stops growing altogether before it reaches the female gametophyte (Moran Lauter et. al. 2017; Lu et al. 2014; Lausser et al. 2010).

The *Ga1*, *Ga2*, and *Tcb1* loci of the genus *Zea* mediate cross incompatibility through male (pollen-expressed) and female (silk-expressed) components of the functional haplotype which is designated using the *-S* notation. The three loci are functionally independent, but genetically similar. The silk component of the *-S* haplotype creates a barrier that excludes any pollen carrying the nonfunctional or the null haplotype (*ga1*, *ga2*, or *tcb1*). Pollen of the *-S* haplotype can overcome the exclusion barrier present in silks carrying the same haplotype. The pollen of these haplotypes can successfully fertilize silks of the null haplotype as well. Therefore, cross-incompatibility is unidirectional. A third haplotype is designated the *-M* haplotype because it only has the male functionality of the loci. Pollen of the *-M* haplotype (*Ga1-M*, *Ga2-M*, or *Tcb1-M*) like the *-S* haplotype pollen can overcome the barrier to pollen tube growth in the silk of the corresponding *-S* haplotype but the plants of this haplotype lack the female function, and therefore their silks can be successfully fertilized by pollen from the null haplotype.

Both male and female components of the three cross-incompatibility loci encode enzymes called pectin methylesterases (PMEs). PMEs modify pollen tube cell walls by hydrolyzing methyl ester groups on homogalacturonan polymers, leading to release of methoxy groups as methanol. Free protons are also released in the process, thereby reducing the local pH. Free carboxyl groups on de-esterified pectin may combine with surrounding Ca^2+^ ions to form pectate gels, leading to cell wall stiffening (Catoire et al 1998). A drop in the pH in the microenvironment because of proton release during de-esterification can activate several downstream enzymes such as polygalacturonases and pectin lyases that loosen the cell wall and promote its extension to help elongate the pollen tube. (Moustacas et al 1991). Therefore the de-esterification reaction alters the extensibility and flexibility of the growing pollen tube.

It has been established in several studies that pollen PMEs influence pollen tube growth and thus play a crucial role in sexual reproduction in plants. VANGUARD1, a pectin methylesterase was found to promote pollen tube growth in Arabidopsis (Jiang et al. 2005). Subsequently, another PME called AtPPME1 was demonstrated to play a role in pollen tube growth (Tian et al. 2006). Similarly, Bosch et al. (2005) identified a tobacco PME, named NtPPME1 that is specifically expressed in pollen and pollen tubes.

In maize, the pollen-expressed determinants of the three loci *i.e.,,* ZmGA1P, ZmGA2P and TCB1-m are all PMEs and ZmGA1F, ZmGA2F and TCB1-F are the corresponding silk-expressed PMEs. Pollen tube growth in these crosses is controlled by interaction of the male gametophyte and female sporophyte and depends on the haplotypes of the interacting partners.

Because all three maize cross incompatibility loci encode PMEs and PME activity is known to be important in controlling pollen tube growth, it seems likely that PME activity is related to pollination success in these systems, however direct measurements of PME activities in reproductive tissues of these genotypes have not been reported. For this reason, we assessed total PME activity in pollinated silks of compatible and incompatible pollinations and examined whether activity levels are related to the success of fertilization. We chose the *Ga1* and *Ga2* cross incompatibility loci for the present study as they represent more diverged loci as compared to *Ga1* and *Tcb1,* which diverged relatively recently (Lu et al. 2019; Bapat et al. 2023) and are likely to function very similarly. A comparison of total PME activity in crosses between different haplotypes of the *Ga1* and *Ga2* systems may provide more insights into tissue and haplotype-specific roles of pectin methylesterase enzymes in cross incompatibility mechanisms.

## Materials and methods

### Experimental design

Separate experiments were carried out to examine total PME activity in the *Ga1* and *Ga2* systems. Each experiment involved statistical comparison of total PME activity in unpollinated silk, ungerminated pollen and pollinated silks from plants with the haplotypes *Ga1-S*, *ga1*, for the *Ga1* locus and analogous haplotypes at the *Ga2* locus. Each haplotype was examined in at least two genetic backgrounds (Refer Table 1 and Supplementary Table S1). Pollinated silk assays were divided into groups defined by the haplotypes used to make the crosses as summarized in Table 1. Pollination groups are further divided into compatible and incompatible pollination types. Some pollination groups, for example *Ga1-S* x *ga1* resulted in incompatible crosses, while others for example *ga1* x *Ga1-S* resulted in compatible crosses. Pollinated silk tissues were collected in five growing seasons for *Ga1* haplotypes and in three growing seasons for *Ga2* haplotypes. Summer growing seasons were carried out at the ISU Agronomy Farm, near Boone, Iowa, USA, while other growing seasons were carried out in a greenhouse. For both *Ga1* and *Ga2* studies, between 10 to 20 crosses per pollination group per growing season were made and pollinated silks from these crosses were examined for total PME activity as described below. All genotypes used in the study were homozygous for their respective allele status (Table S1).

**Table 1:** Summary of experimental design for analysis of pollinated silks

Silk haplotype	Pollen haplotype	Pollination groups	Pollination type	Growing seasons	*N*	Genotypes involved
*Ga1-S*	*Ga1-S*	*Ga1-S* × *Ga1-S*	Compatible	Summer 2020, Spring 2021, Summer 2021, Fall 2021, Spring 2022	64	Ga1-401D(*Ga1-S/Ga1-S*), Hp301(*Ga1-S/Ga1-S*), PHG35 (*ga1/ga1*),USDA Blue (ga1/ga1)
*ga1*	*Ga1-S*	*ga1* × *Ga1-S*	Compatible	Summer 2020, Spring 2021, Summer 2021, Fall 2021, Spring 2022	63	
*ga1*	*ga1*	*ga1* × *ga1*	Compatible	Summer 2020, Spring 2021, Summer 2021, Fall 2021, Spring 2022	60	
*Ga1-S*	*ga1*	*Ga1-S* × *ga1*	Incompatible	Summer 2020, Spring 2021, Summer 2021, Fall 2021, Spring 2022	67	
*Ga2-S*	*Ga2-S*	*Ga2-S* × *Ga2-S*	Compatible	Spring 2021, Summer 2021, Spring 2022	42	Ga2 104–3(*Ga2-S/Ga2-S*), MGSC-Ga2-S(511L)(*Ga2-S/Ga2-S*), MGSC -Ga2-S(511 M) (*Ga2-S/Ga2-S*), PHG35(*ga1/ga1*), USDA Blue (*ga1/ga1*)
*ga2*	*Ga2-S*	*ga2* × *Ga2-S*	Compatible	Spring 2021, Summer 2021, Spring 2022	44	
*ga2*	*ga2*	*ga2* × *ga2*	Compatible	Spring 2021, Summer 2021, Spring 2022	37	
*Ga2-S*	*ga2*	*Ga2-S* × *ga2*	Incompatible	Spring 2021, Summer 2021, Spring 2022	41	

### Plant tissue collection

Mature tassels were bagged on the evening before pollination and ear shoots were covered prior to silk emergence. Pollinations were done between 9:00 to 12:00 P.M. Approximately 8 cm of pollinated silks were removed between 4 and 6 h after pollination for crosses involving *Ga1* haplotypes. Collection of silk tissues for crosses of the *Ga2* haplotypes was done between 2 and 4 h after pollination. Collection times were based on reports of pollen tube growth rates for the two systems under study (Moran Lauter et al. 2017; Wang et al. 2018). The goal was to harvest at time points when there is a difference in the pollen tube growth rates between compatible and incompatible crosses. Fresh shed pollen and unpollinated silks were collected between 9:00 A.M to noon and frozen in liquid N_2_. Frozen tissues were processed and analyzed for pectin methylesterase activity within 24 h of collection.

### Preparation of crude extracts from silk and pollen

Pollinated and unpollinated silk tissues were ground in liquid nitrogen using a pestle and mortar. 2.5 g of ground silk tissue was thawed in 5 ml ice-cold extraction buffer (200 mM sodium phosphate-500 mM NaCl, pH 6.6) for 5 min. The samples were then vortexed for 30 s and incubated in an orbital shaker at 250 rpm for 1 h at 4 °C. The extracts were clarified by centrifugation at 20,000 × g for 30 min at 4 °C. The supernatant was pipetted into microcentrifuge tubes and stored in ice for analysis. The remaining supernatant was frozen for re-analysis if required. 250 mg of pollen was ground in liquid nitrogen in a pestle and mortar and vortexed briefly in 5 ml ice-cold extraction buffer, and the remaining steps for extraction were followed as for the silk tissues. The total protein concentration of all samples was estimated using the Bradford protocol (Bradford 1976). Bovine serum albumin was used as the standard.

### PME activity assay

Silk and pollen extracts were assayed for PME activity using the modified Fluoral-P assay (Klavons and Bennett 1986; Wojciechowski and Fall 1996). Briefly, the reaction mixture was prepared by mixing 0.5% pectin (from citrus fruits, > 85% esterification, Sigma-Aldrich, P9561) in phosphate buffer, 200 mM sodium phosphate buffer (pH 6.6) and 0.0025 U**/**μl alcohol oxidase (from *P. pastoris*, Sigma, A2404). 20 μl of crude silk or pollen extracts were added to the reaction mixture and the reaction was incubated for 10 min at 25 °C. After incubation, 4 μg/μl (final concentration) 4-Amino-3-penten-2-one (Fluoral-P) (Sigma-Aldrich, 691,003) is added to the sample mix and incubated for another 5 min. Formaldehyde produced due to alcohol oxidase interacts with Fluoral-P to create a fluorescent product which was measured using a Tecan 96-well fluorimeter with 405 nm excitation and 510 nm emission filters. The amount of methanol released was estimated using standards with known methanol concentrations (0 to 20mM). Pectinesterase (from orange peel, Sigma-Aldrich, P5400) was used as a positive control. The negative control contained distilled water in place of PME. PME activity was expressed as nmol of methanol min^−1^ μg protein ^−1^. For a given tissue, the same mass of tissue was used in each assay so PME activity data is comparable within tissues, but not across tissues.

### Multiple sequence alignments of PME proteins in maize

*Daucus carota* PME protein sequence (P83218) was downloaded from UnitProtKB. *Ga1*, *Ga2* and *Tcb1* associated PME sequences and ZmPME10-1 protein sequence were downloaded from MaizeGDB. All other putative maize PMEs were downloaded from GenBank. Multiple sequence alignments were constructed using the ClustalO command line program. Active site residues in *D. carota* PME and corresponding substitutions in maize PMEs were identified in the alignment using the *D. carota* PME as the reference sequence.

### Data analysis and statistical tests

Data analysis and statistical tests were performed using R. Graphs were generated with the R package ggplot2. Data used in statistical models were evaluated using Shapiro Wilk and Levene’s tests to check for normality and homoscedasticity assumptions respectively. After modeling, Q-Q plots and residual vs fitted plots were generated to visualize the distribution of the data and check for unequal variances in the datasets. (Figs. S1-S4).

Factorial fixed effect ANOVA models using PME activity as the response variable were fit using least squares modeling to estimate the significance and magnitude of experimental effects. Data from *Ga1* and *Ga2* pollinated silk, unpollinated silk and ungerminated pollen tissues were modeled separately. The ANOVA models for ungerminated pollen and unpollinated silk tissue included the effects of haplotype, growing season, and haplotype x growing season (Table 2 and 4). The effect of interest was haplotype, which allowed testing for significant variation among the haplotypes included in the study.

**Table 2:** Factorial analysis of variance table for *Ga1* pollen haplotypes

Factorial ANOVA					
	Df	Sum Sq	Mean Sq	*F* value	Pr(> *F*)
Haplotype	1	0.212	0.21202	10.239	0.00249**
Growing season	4	1	0.24999	12.073	8.65E-07***
Haplotype × Growing season	4	0.208	0.052	2.511	0.05452
Residuals	46	0.9525	0.02071		

Table 6 and 7 summarize the models used for *Ga1* and *Ga2* pollinated silks, respectively. The main effects in the models included pollen haplotype, silk haplotype and the growing season. All possible interactions between the two main effects i.e.,, male and the female haplotypes in a cross was also tested, as well as a three-way interaction effect. The silk haplotype × pollen haplotype effect is of particular interest, because it tests for significant variation among pollination groups. Significance of this effect would support the hypothesis that total PME activity is determined by an interaction between the female and male factors of the locus being studied. This interaction would reflect the well characterized genetic interaction between the haplotypes and may also imply a biochemical interaction between proteins. The silk haplotype × pollen haplotype effect was significant in the *Ga1* study of pollinated silk (Table 6, Table S2, Fig. S5), but not in the *Ga2* study of pollinated silk (Table 7). The effect of growing season is included to control for environmental impacts on total PME activity. Tukey’s post-hoc HSD test (significance level = 0.05) was used for comparison whenever *F* values of the effects of interest were significant.

## Results and discussion

### PME activity in silk and pollen prior to pollination

**Fig. 1:**
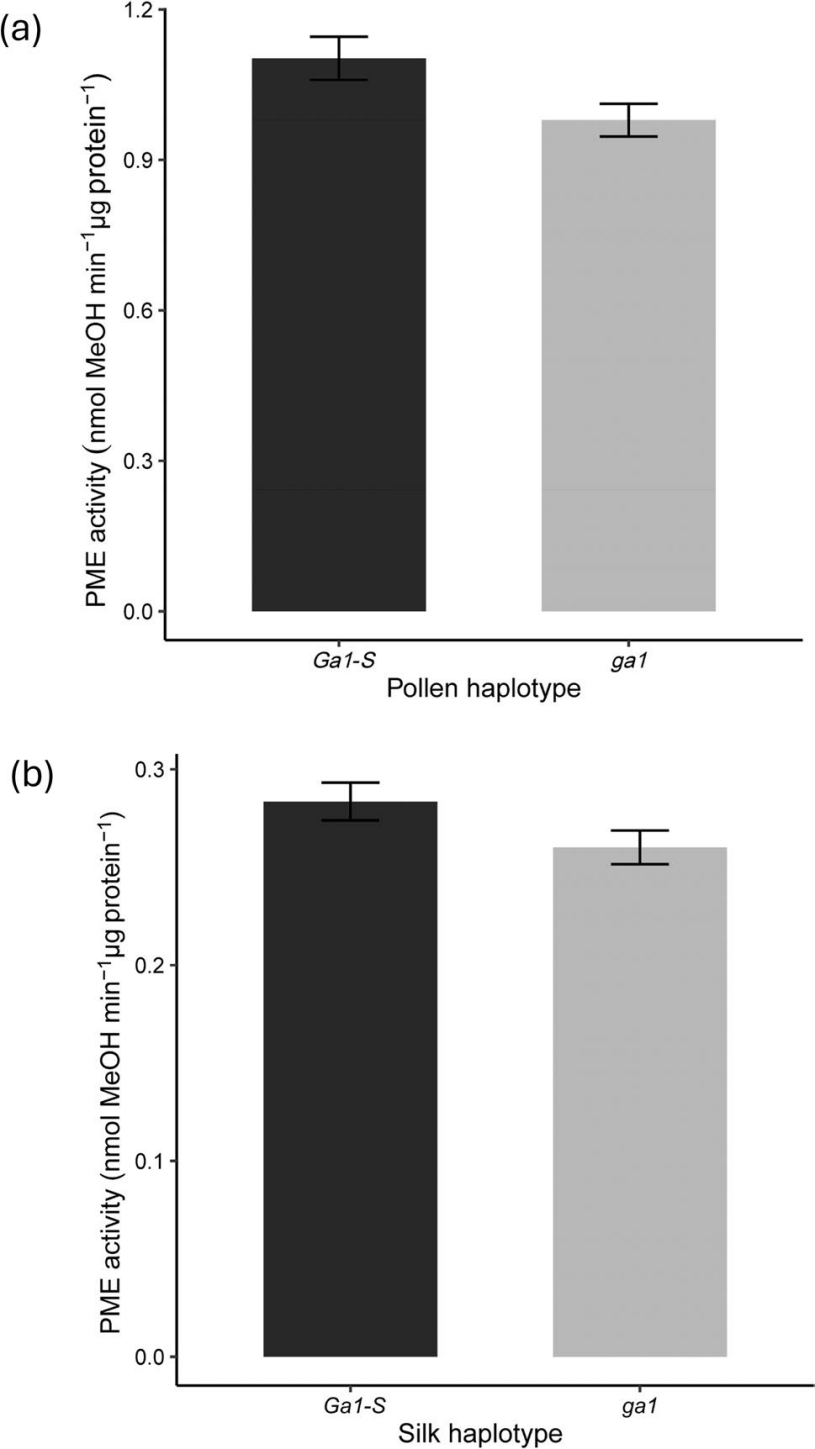
Mean PME activity in pollen and silk tissues of the *Ga1* haplotypes. Bar plots with error bars represent means ± SE of haplotypes. **a** PME activity in ungerminated pollen of *Ga1-S* and *ga1* haplotypes **b** PME activity in unpollinated silks in the *Ga1-S*, and *ga1* haplotypes

To better understand the role of total PME activity in cross incompatibility we measured PME activity in ungerminated pollen and unpollinated silk tissues from the *–S* and the null haplotypes of the *Ga1* and *Ga2* systems. In ungerminated pollen of the *Ga1* system, PME activity in *Ga1-S* pollen haplotypes was significantly higher than *ga1* pollen haplotypes. (Table 2, Fig. 1a, Table S2a). PME activity was not significantly different between *Ga1-S* and *ga1* haplotypes in unpollinated silks (Table 3, Fig. 1b, Table S2b).

**Table 3:** Factorial analysis of variance table for *Ga1* silk haplotypes

Factorial ANOVA					
	Df	Sum Sq	Mean Sq	*F* value	Pr(> *F*)
Haplotype	1	0.00766	0.007665	3.111	0.0844
Growing season	4	0.00956	0.00239	0.97	0.433
Haplotype × Growing season	4	0.00385	0.000962	0.391	0.8142
Residuals	46	0.11333	0.002464		

**Fig. 2:**
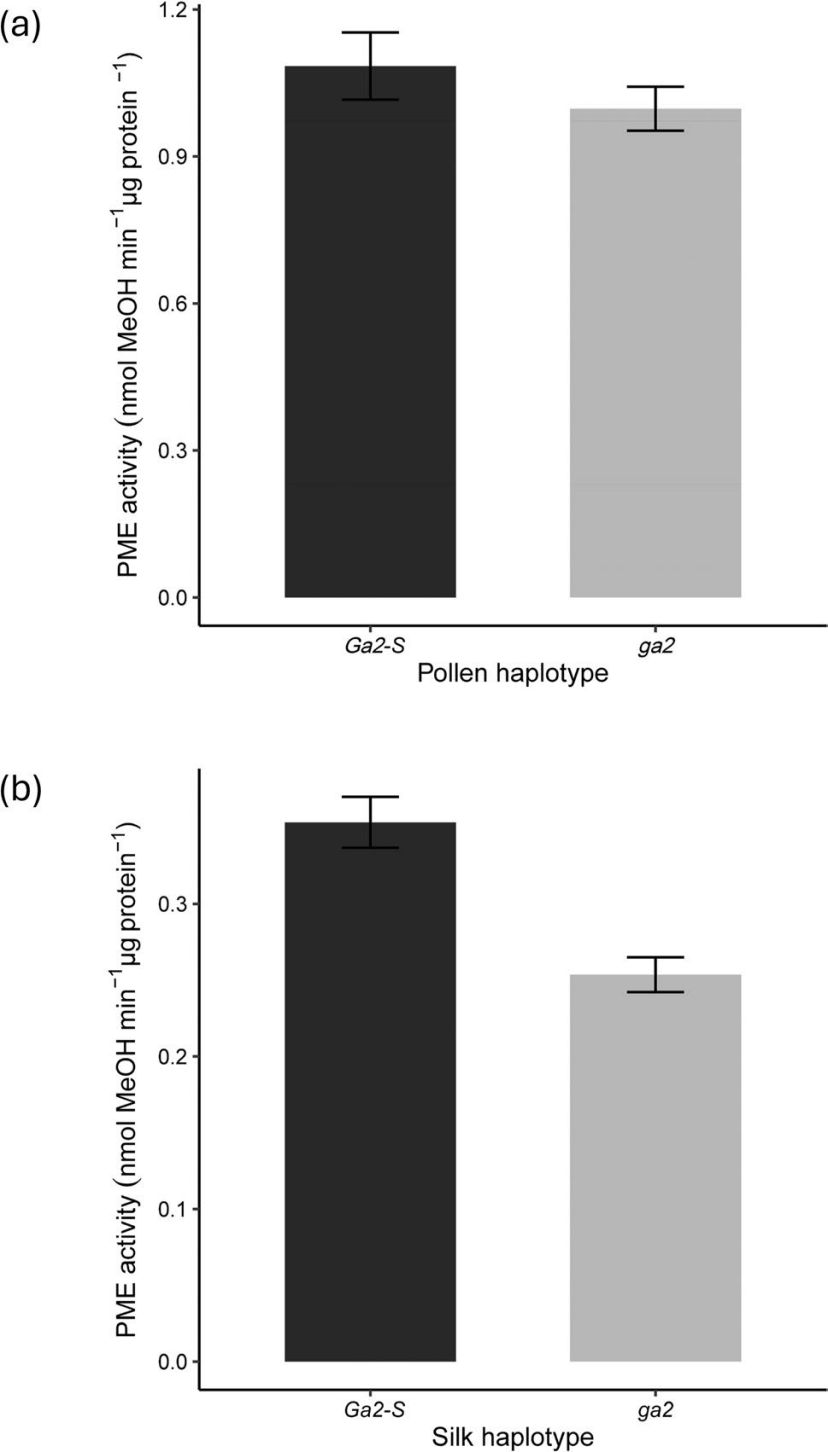
Mean PME activity in pollen and silk tissues of *Ga2* haplotypes. Bar plots with error bars represent means ± SE of haplotypes **a** PME activity in ungerminated pollen of *Ga2-S*, and *ga2* haplotypes **b** PME activity in unpollinated silks in the *Ga2-S* and *ga2* haplotypes

In ungerminated pollen of the *Ga2* system, no significant differences were observed in PME activity between the *–S* and the null haplotypes (Table 4, Fig. 2a). In unpollinated silks, the *Ga2-S* haplotypes had significantly higher levels of PME activity than the *ga2* haplotypes (Table 5, Fig. 2b, Table S3).

**Table 4:** Factorial analysis of variance table for *Ga2* pollen haplotypes

Factorial ANOVA					
	Df	Sum Sq	Mean Sq	*F* value	Pr(> *F*)
Haplotype	1	0.0587	0.0587	3.001	0.0956
Growing season	2	0.1748	0.0874	4.469	0.0219*
Haplotype × Growing season	2	0.8816	0.4408	22.538	2.54E-06***
Residuals	25	0.489	0.0196

**Table 5:** Factorial analysis of variance table for *Ga2* silk haplotypes

Factorial ANOVA					
	Df	Sum Sq	Mean Sq	*F* value	Pr(> *F*)
Haplotype	1	0.08461	0.08461	24.371	3.30E-05***
Growing season	2	0.00647	0.00324	0.932	0.406
Haplotype × Growing season	2	0.00861	0.00431	1.24	0.305
Residuals	28	0.09721	0.00347		

Signif. Codes: 0 ‘***’ 0.001 ‘**’ 0.01 ‘*’ 0.05 ‘.’ 0.1 ‘ ’ 1

These observations suggest that the presence of the silk-expressed PME gene of the *Ga2* system is related to the total PME activity in unpollinated silks, while the presence of the pollen-expressed PME is related to the total PME activity in pollen from the *Ga1* system.

### PME activity in pollinated silks of plants with different Ga1 haplotypes

**Fig. 3:**
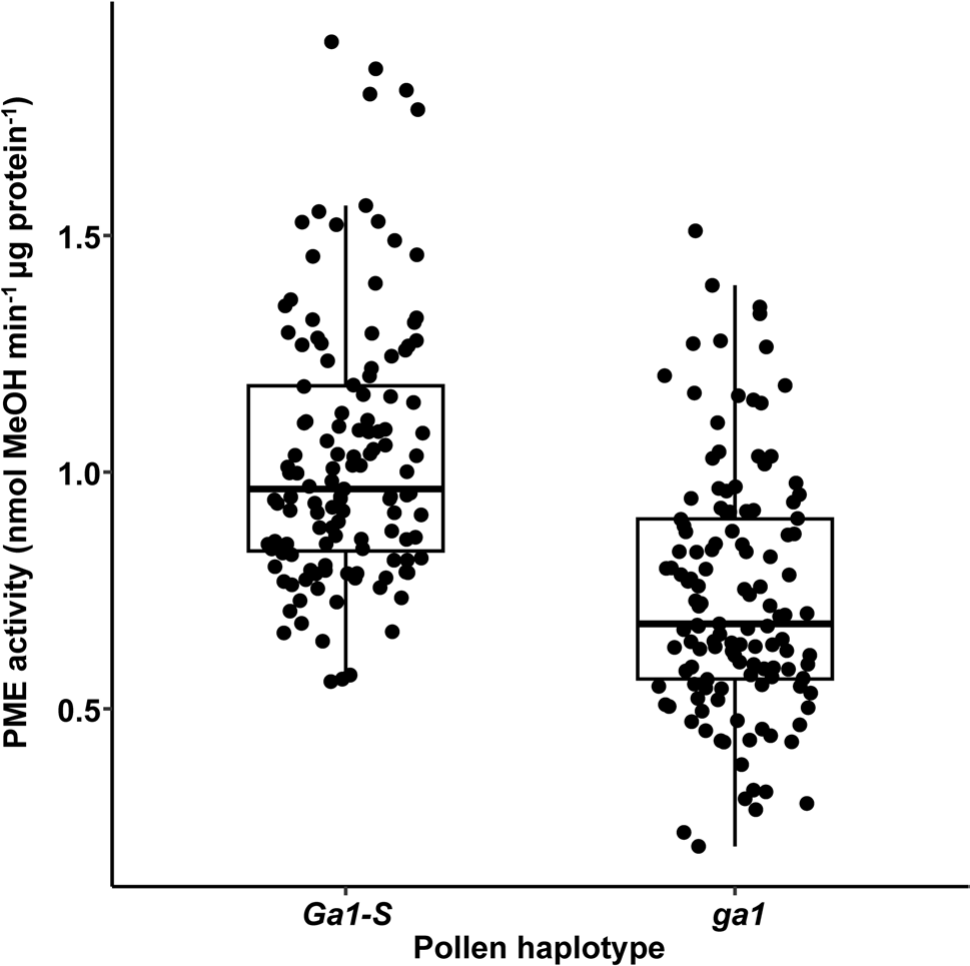
Total PME activities by pollen haplotype in the *Ga1* study

**Fig. 4:**
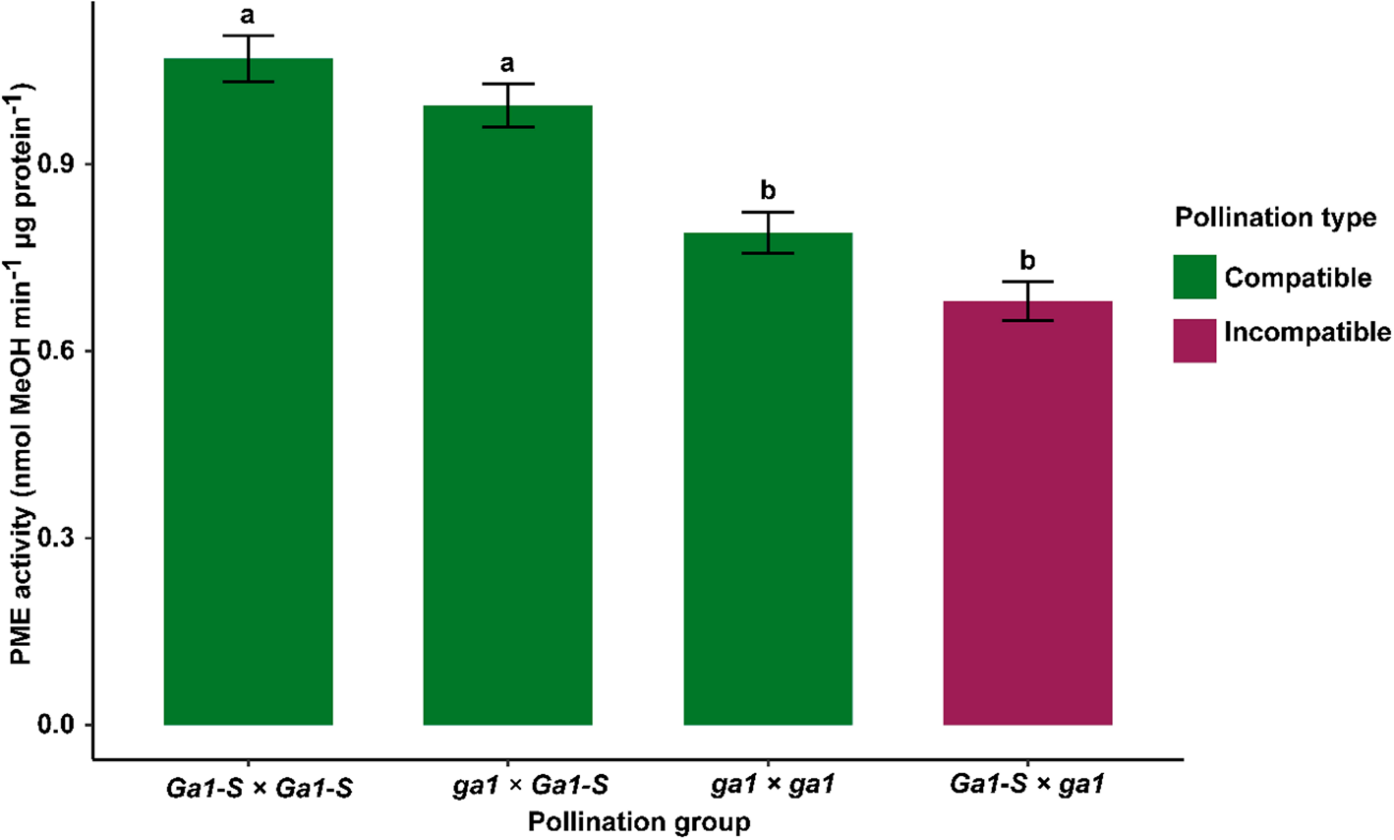
Mean total PME activity in crosses involving *Ga1-S* and *ga1* haplotypes in the *Ga1* study. Bar plots with error bars represent means ± SE of pollination groups. The means of bars labeled ‘**a**’ differ from those labeled ‘**b**’, according to analysis of variance followed by multiple comparison of means using Tukey’s HSD test (*p* < 0.05)

Next, we set out to examine PME levels in pollinated silks in different combinations of silk and pollen haplotypes. This was done by making four groups of crosses, three of which were predicted to result in compatible crosses (*ga1* x *ga1*, *Ga1-S* x *Ga1-S*, and *ga1* x *Ga1-S*) while the fourth group (*Ga1-S* x *ga1*) was predicted to result in incompatible crosses. The growing season-wise and genotype-wise total PME activity in the crosses is depicted in Fig. S6 and S7, respectively. The main effect of pollen haplotype was significant, while the main effect of the silk haplotype was not (Table 6, Fig. 3). PME activity was significantly higher in crosses involving the *Ga1-S* pollen haplotype as compared to crosses in involving the *ga1* pollen haplotype. The significant effect of the pollen haplotype establishes a correlation between the *Ga1* pollen PME and PME activity in pollinated silks. There is also a significant interaction effect between the female haplotype × male haplotype (Table 6, Table S4). The significant effect of the interaction between silks and pollen haplotype on total PME activity provides a biochemical parallel for the idea advanced by several groups (reviewed in Lu et al. 2020) that a genetic interaction between haplotypes determines pollination outcomes (Fig. 4). We tested the hypothesis that the genetic interaction between *Ga1* haplotypes is paralleled by biochemical interaction between the haplotypes that results in a change in PME activities that is correlated with the success or failure of pollination. The data presented in Fig. 4 do not support the conclusion that pollination outcomes are dependent on the total PME activity levels in pollinated silks of the *Ga1* system. However, because this conclusion is based in part on a lack of significance in the comparison between the means of the *ga1* x *ga1* and the *Ga1-S* x *ga1* crosses, an experiment with more statistical power may support a different conclusion.

**Table 6:** Factorial analysis of variance in *Ga1* study of pollinated silk

Factorial ANOVA					
	Df	Sum Sq	Mean Sq	*F* value	Pr(> *F*)
Pollen haplotype	1	5.531	5.531	83.613	< 2.00E-16***
Silk haplotype	1	0.039	0.039	0.586	0.444673
Growing season	4	1.618	0.404	6.114	0.000107***
Silk haplotype × Pollen haplotype	1	0.633	0.633	9.572	0.002216**
Pollen haplotype × Growing season	4	0.113	0.028	0.425	0.790273
Silk haplotype × Growing season	4	0.288	0.072	1.09	0.362319
Silk haplotype x Pollen haplotype × Growing season	4	0.189	0.047	0.713	0.58369
Residuals	234	15.479	0.066

### PME activity in pollinated silks of plants with different Ga2 haplotypes

**Fig. 5:**
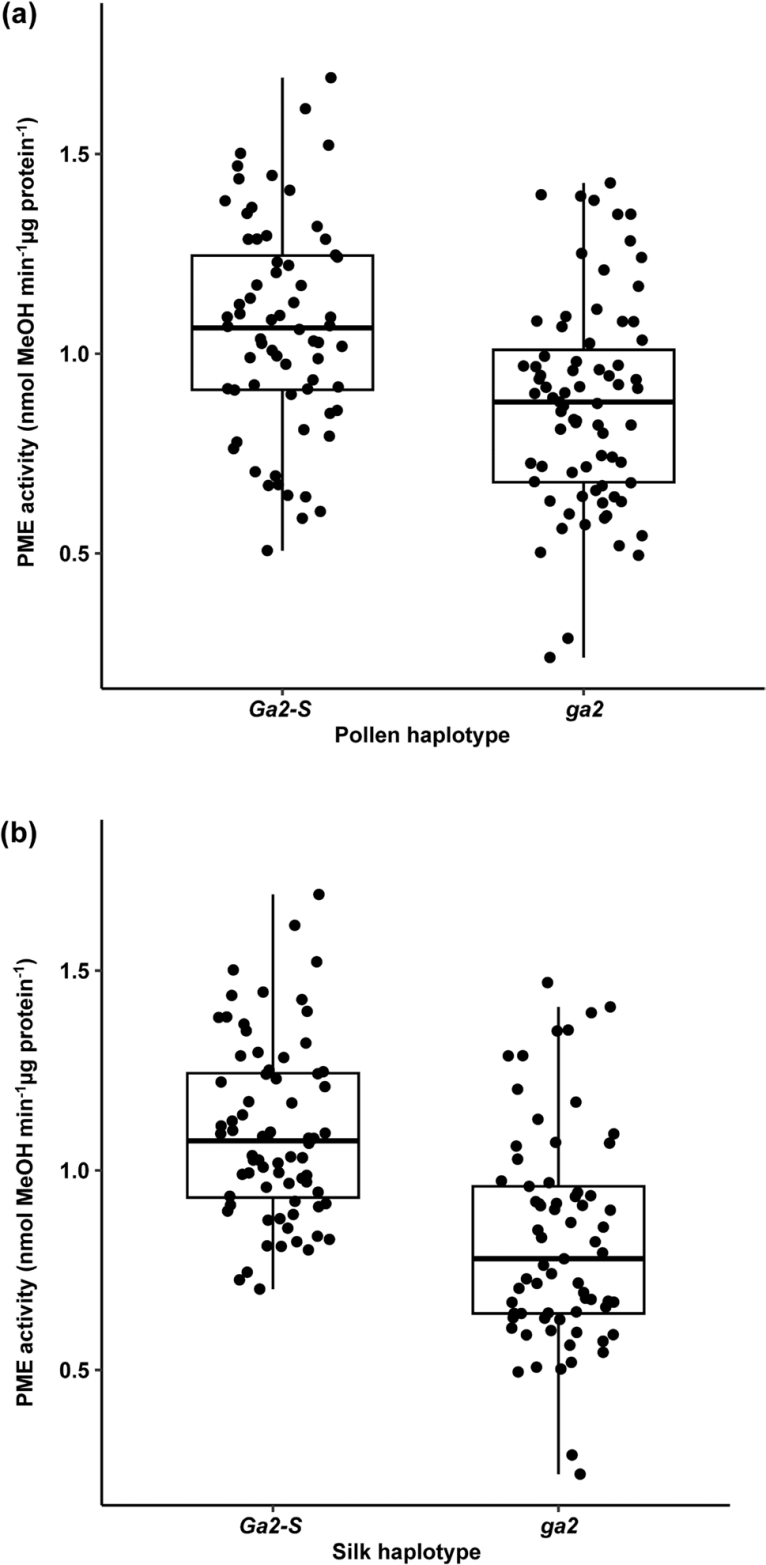
Haplotype and tissue specific total PME activity in the crosses *Ga2* study. **a** Differences in total PME activity by pollen haplotype. **b** Differences in total PME activity by silk haplotype

We next applied a similar approach to characterize total PME activity in pollinated silks in crosses among plants with different haplotypes at the *Ga2* locus. Like *Ga1*, the main effect of pollen haplotype was significant (Table 7), with crosses having the *Ga2-S* pollen haplotype having higher PME activity than those with the *ga2* haplotype (Fig. 5a). In addition, the main effect of the silk haplotype was significant in crosses among different haplotypes of the *Ga2* system, again showing a higher PME activity in crosses with *Ga2-S* as the silk haplotype as compared to crosses with *ga2* as silk haplotype. (Table 7 and Fig. 5b). The interaction between silk haplotype and pollen haplotype was not significant in the *Ga2* system (Table 7), so we could not make a meaningful comparison among Ga2 crosses, analogous to the comparison made in Fig. 4 for Ga1 crosses. Figs. S8 and S9 show growing season-wise and genotype-wise total PME activities in *Ga2* crosses, respectively. To summarize, the only experiment that yielded statistically significant results in both the *Ga1* and *Ga2* systems was examination of the main effect of the pollen haplotype, and in this experiment the observations were consistent between the two systems. While these observations do not provide a complete explanation of the molecular mechanisms controlling the process of cross incompatibility, the control of PME activity levels in pollinated silk by factors in pollen may be part of this mechanism. Future experiments would benefit from more replication to increase the statistical power of the experiments. Additional genotypic replications would aid in ruling out the influence of genotypic factors on PME activity.

**Table 7:** Factorial analysis of variance table for *Ga2* study of pollinated silk

Factorial ANOVA					
	Df	Sum Sq	Mean Sq	*F* value	Pr(> *F*)
Pollen haplotype	1	1.268	1.2683	25.29	1.62E-06***
Silk haplotype	1	2.522	2.5223	50.296	7.79E-11***
Growing season	2	0.066	0.0332	0.661	0.5179
Silk haplotype × Pollen haplotype	1	0.026	0.0261	0.521	0.4718
Pollen haplotype × Growing season	2	0.091	0.0457	0.911	0.4048
Silk haplotype × Growing season	2	0.36	0.18	3.589	0.0304*
Silk haplotype x Pollen haplotype × Growing season	2	0.06	0.0298	0.594	0.5536
Residuals	129	6.469	0.0502		

Signif. Codes: 0 ‘***’ 0.001 ‘**’ 0.01 ‘*’ 0.05 ‘.’ 0.1 ‘ ’ 1

### Unique active site substitutions that differ between pollen and silk PMEs are conserved across all three cross incapability systems

**Fig. 6:**
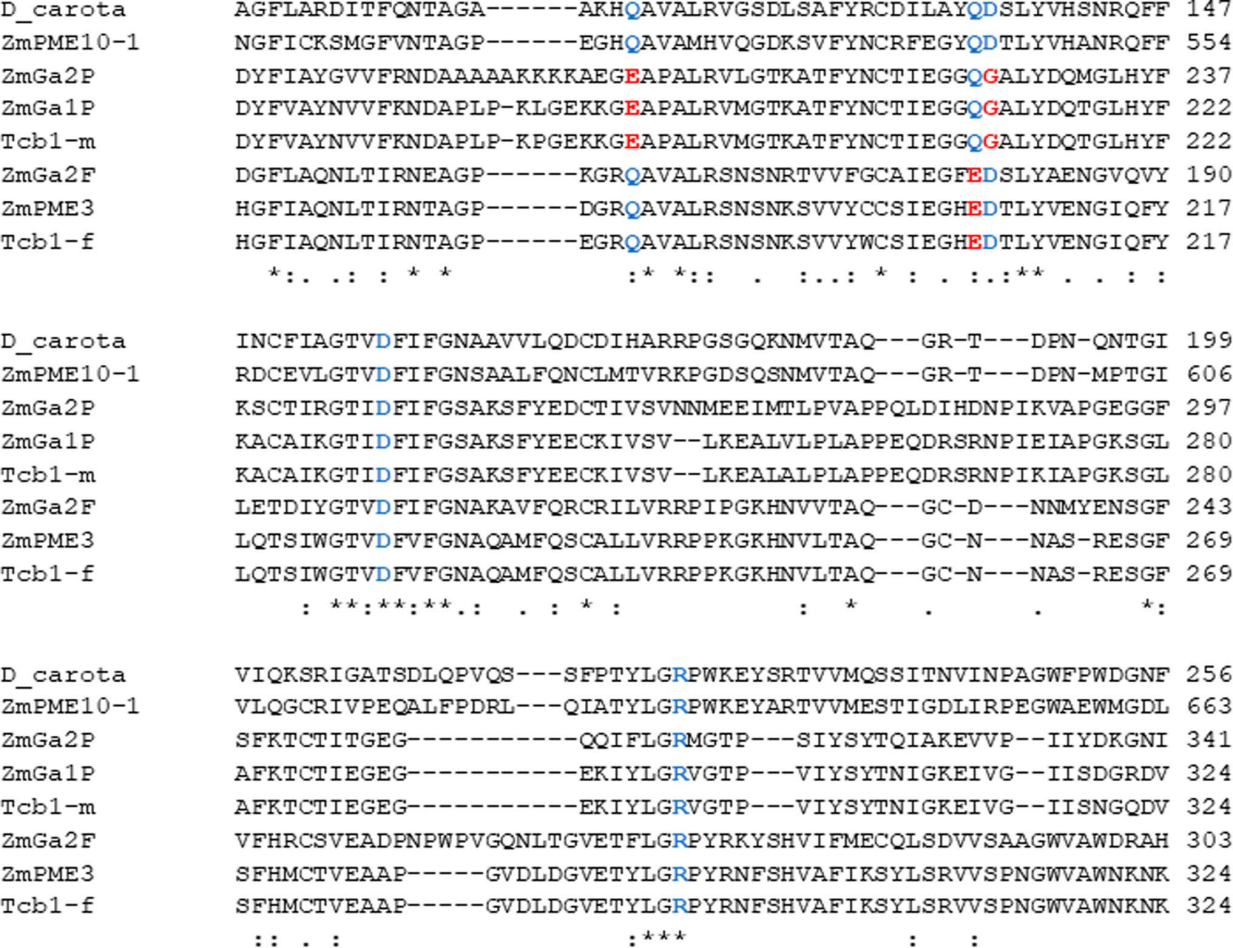
Partial multiple sequence alignment of *D. carota* PME, ZmPME10-1 and male and female PMEs of the three gametophytic incompatibility loci. The active site amino acid residues in the *D. carota* PME and the corresponding amino acids residues in all other PMEs have been marked in blue. The active site substitutions have been marked in red

It is not clear if the variation in total PME levels observed in this study is due to a direct contribution of PME activity by the PMEs encoded by the *Ga1* and *Ga2* loci, or if these loci serve to regulate PME activity through interactions with other PMEs. Physical interactions between *Ga1* and *Ga2* encoded PMEs and ZmPME10-1 (a pollen PME present in all maize pollen) have been demonstrated in vitro (Zhang et al. 2018; Chen et al. 2022). In addition, Chen et al. (2022) have presented an alignment of cross incompatibility associated PME active-site residues and pointed out that “various mutations” occur in the active site residues “suggesting they may not possess in vivo PME activities, and yet may directly interact with other active PMEs” We carried out further examination of the active site residues of PMEs involved in the cross-incompatibility systems of maize. The active site of the *D. carota* PME is composed of Asp113, Gln135, Asp136, Gln157 and Arg225 amino acid residues (Johansson et al. 2002). These amino acids are highly conserved in all maize PMEs not involved in cross-incompatibility (Fig. S10). Figure 6 is a partial multiple sequence alignment showing the active site residues of *D. carota* PME, male and female function PMEs of the three gametophytic cross incompatibility loci, *Ga1*, *Ga2* and *Tcb1* and ZmPME10-1. As observed by Chen et al. (2022), the alignment shows that active site amino acid substitutions are present in all cross incompatibility PME proteins. While the active site residues identified in the *D. carota* sequence are conserved in the majority of PMEs in maize including ZmPME10-1, the silk PMEs of cross incompatibility loci i.e.,, ZmPME3, ZmGa2F and TCB1-F all share a Gln → Glu substitution at position 135 of the *D. carota* sequence. All the three pollen PMEs i.e.,, ZmGA1P, ZmGA2P and TCB1-M have an Asp → Gly change at position 136 of the *D. carota* PME. The male function PMEs also have a Gln → Glu change at position 113 of the *D. carota* active site. The active site substitutions in PMEs encoded by maize cross incompatibility loci are not only unique among maize PMEs, but they differ among the pollen and silk function PMEs, and these differences are conserved across the three cross incompatibility systems. Conservation across all three cross incompatibility loci is a result of the close evolutionary relationship among the cross-incompatibility systems of maize (Bapat et al. 2023). Given that all three substitutions change the charge of the amino acids present, they are likely to impact PME activity. They may reflect unique functions of these PMEs relative to other PMEs encoded in maize, while the differences between the pollen and silk function PMEs suggests that they may use different mechanisms to regulate the process of pollen tube growth. Conservation of these changes across Ga1 and Ga2 may suggest that these two systems have functional similarities.

There is a possibility that the PMEs of maize incompatibility systems function in a regulatory capacity that does not depend on their PME activity (Zhang et al. 2018; Chen et al. 2022). In this paper, we observed differences in total PME activity between tissues carrying functional and non-functional alleles of the *Ga1* and *Ga2* systems. This work provides additional support to the hypothesis that the cross-incompatibility loci of maize function by altering PME activity levels in pollinated silk (Zhang et al. 2018; Moran Lauter et al. 2017; Lu et al. 2020). It is possible that these differences are due to the activities of enzymes encoded by the PMEs of these systems. An alternative is that the differences in total PME activity results from differential regulation of endogenous PME activity by the PMEs encoded by the maize cross incompatibility loci.

## Conclusion

PMEs play an important role in pollen tube growth. The present study involved in vitro assessment of total PME activity in reproductive tissues of plants with different haplotypes of the *Ga1* and *Ga2* cross incompatibility systems. We found that the presence of the silk function PME gene of the *Ga2* system is related to the PME activity in silks, while the presence of the pollen-function PME is related to the PME activity in pollen from the *Ga1* system. We also examined total PME activity levels in pollinated silks from crosses involving different combinations of haplotypes and discovered that the pollen haplotype *Ga1-S* has a significant effect on total PME levels. Further, the interaction between the pollen and silk haplotypes of *Ga1* significantly affects total PME levels, however we were unable to detect a significant relationship between PME activity levels and fertilization success. As has been observed (Chen et al. 2022), the pollen and silk PMEs encoded by cross incompatibility systems have amino acid substitutions at conserved active site residues that may impact the PME activity of these proteins. Interestingly, the active site mutations of the pollen-expressed and silk-expressed PMEs of the cross incompatibility systems are different, and this difference is conserved across the *Ga1*, *Ga2* and *Tcb-1* systems. These active site amino acid substitutions may result in a functional distinction between the molecular mechanisms by which pollen and silk function PMEs encoded by cross incompatibility loci regulate pollen tube growth. This work provides biochemical support for the widely held hypothesis that cross incompatibility loci of maize function through modulation of PME activity levels. It is not clear if this modulation is due to regulation of the activities of other PMEs or to the activities of the PMEs encoded by the cross-incompatibility loci or to some combination of the two.

## Supplementary Information

Below is the link to the electronic supplementary material.

Supplementary file1 (PDF 2389 kb)
