# Supplementary material for: Pectin methylesterase activities in reproductive tissues of maize plants with different haplotypes of the Ga1 and Ga2 cross incompatibility systems
Source: Plant Reprod. 2024 May 3;37(4):479–88. doi: 10.1007/s00497-024-00502-0 (PMC11511756; doi:10.1007/s00497-024-00502-0)
Supplement: Supplementary file 1 — Supplementary file1 (PDF 2389 kb) [file 497_2024_502_MOESM1_ESM.pdf]

Pectin methylesterase activities in reproductive tissues of maize plants with different haplotypes of the *Ga1* and *Ga2* cross incompatibility systems

Plant Reproduction

Amruta R. Bapat[1,2] and M. Paul Scott[3]

Affiliations:

1. Interdepartmental Genetics and Genomics Program, Iowa State University, Ames, Iowa, USA 50011
2. Department of Agronomy, Iowa State University, Ames, Iowa, USA 50011
3. USDA Agricultural Research Service, Corn Insects and Crop Genetics Research Unit, 716 Farmhouse Lane, Ames, IA, 50011, USA

Email: paul.scott@usda.gov

Supplementary Information

Table S1 Allele status of genotypes used in the study.

Genotype	Allele status
401-D -W22	*Ga1-S, ga2,tcb1*
Hp301	*Ga1-S,ga2,tcb1*
PHG35	*ga1, ga2,tcb1*
USDA Blue	*ga1,ga2,tcb1*
104-3-W22	*ga1,Ga2-S,tcb1*
MGSC - 511L-W22	*ga1,Ga2-S,tcb1*
MGSC - 511M-W22	*ga1,Ga2-S,tcb1*

Table S2 Tukey's HSD for ungerminated *Ga1* pollen

	Tukey multiple comparison of means			
	95% family-wise confidence level			
$Haplotype				
	diff	lwr	upr	p adj
Ga1S-ga1	0.12314	0.045677	0.200603	0.002492

Table S3 Tukey's HSD for unpollinated *Ga2* silks

	Tukey multiple comparison of means			
	95% family-wise confidence level			
$Haplotype				
	diff	lwr	upr	p adj
Ga2S-ga2	0.09977109	0.05837284	0.1411693	3.3e-05

Table S4 Tukey's HSD test for crosses involving *Ga1-S* and *ga1* haplotypes

Tukey's Multiple comparison of Means<br>95% family wise confidence level				
Pollen haplotype : Silk haplotype (Interaction effect)				
	diff	lwr	upr	p adj
Ga1-S:ga1-ga1:ga1	-0.1086	-0.2268	0.00966	0.0500650
ga1:Ga1-S-ga1:ga1	0.21014	0.09147	0.3288	**0.0000435**
Ga1-S:Ga1-S-ga1:ga1	0.2796	0.16008	0.39912	**0**
ga1:Ga1-S-Ga1-S:ga1	0.31873	0.20491	0.43254	**0**
Ga1-S:Ga1-S-Ga1-S:ga1	0.38819	0.27349	0.50289	**0**
Ga1-S:Ga1-S-ga1:Ga1-S	0.06946	-0.0457	0.18459	0.4030972
Growing season				
	diff	lwr	upr	p adj
Spring 2021-Fall 2021	0.01785	-0.1359	0.17162	0.9977466
Spring 2022-Fall 2021	-0.1376	-0.2882	0.01301	0.0915649
Summer 2020-Fall 2021	0.09233	-0.064	0.24862	0.4837391
Summer 2021-Fall 2021	-0.0782	-0.227	0.07061	0.5998261
Spring 2022-Spring 2021	-0.1554	-0.2886	-0.0223	**0.0130415**
Summer 2020-Spring 2021	0.07448	-0.0651	0.21404	0.585066
Summer 2021-Spring 2021	-0.096	-0.2271	0.03508	0.2628649
Summer 2020-Spring 2022	0.22991	0.09387	0.36596	**0.0000543**
Summer 2021-Spring 2022	0.0594	-0.068	0.18676	0.7027837
Summer 2021-Summer 2020	-0.1705	-0.3046	-0.0365	**0.0050415**

(a)

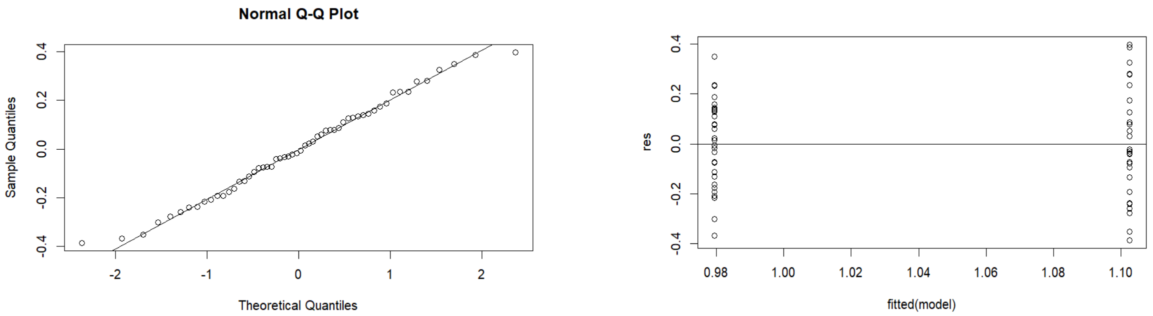

(b)

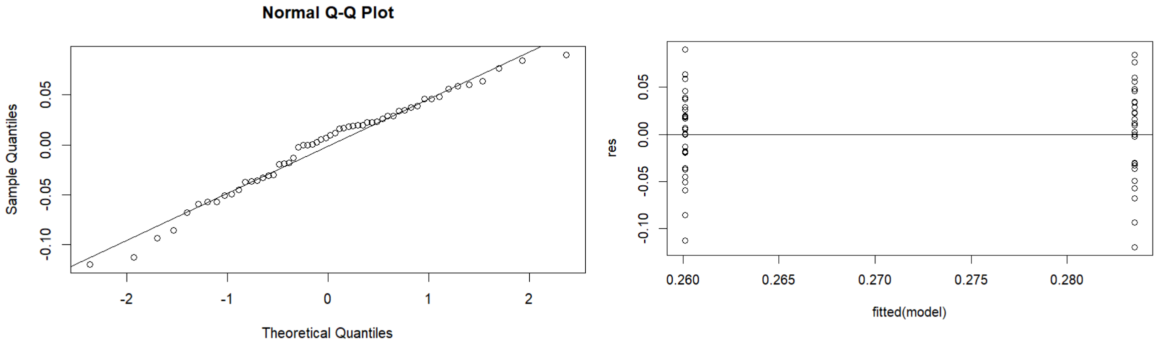

Fig. S1 (a) Q-Q plot and residual vs fitted plot of total PME activity in ungerminated pollen of *Ga1* haplotypes **(b)** Q-Q plot and residual vs fitted plot of total PME activity in unpollinated silks of *Ga1* haplotype

(a)

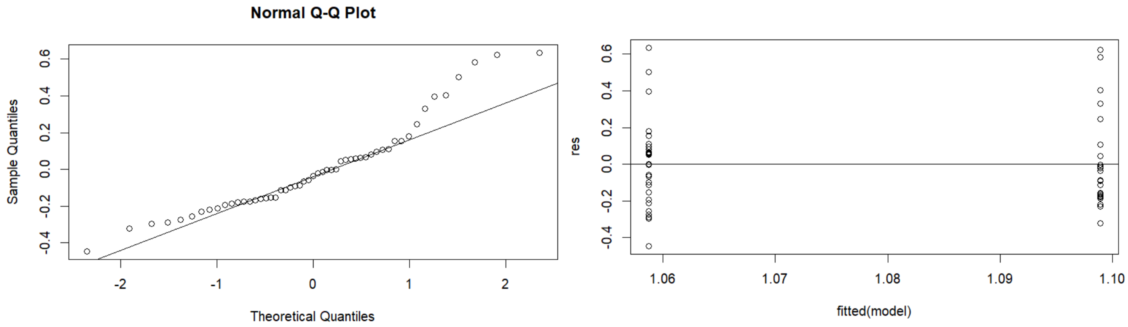

(b)

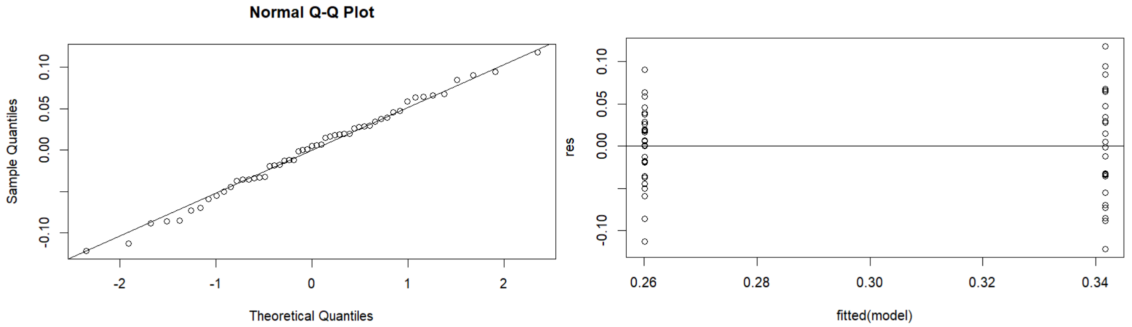

Fig. S2 (a) Q-Q plot and residual vs fitted plot of total PME activity in ungerminated pollen of *Ga2* haplotypes **(b)** Q-Q plot and residual vs fitted plot of total PME activity in unpollinated silks of *Ga2* haplotypes.

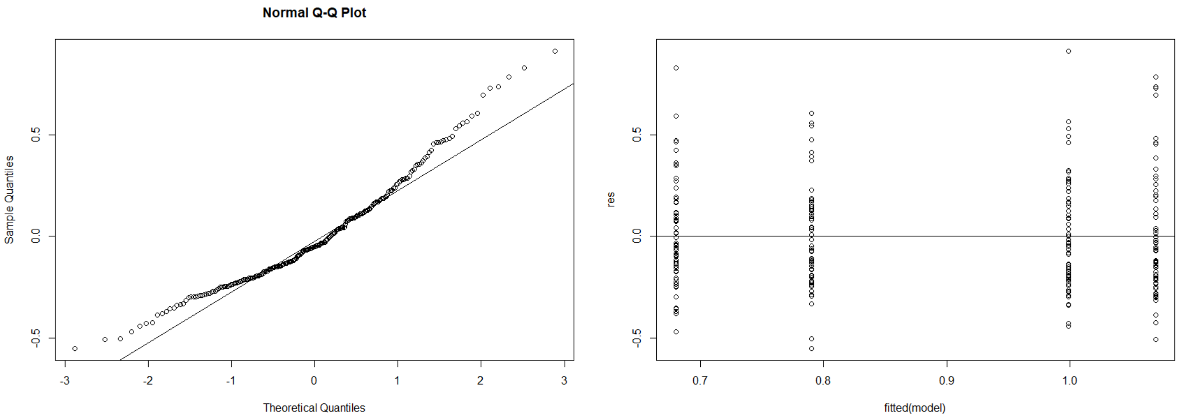

Fig. S3 Q-Q plot and residual vs fitted plot of total PME activity in pollinated silks in the *Ga1* study

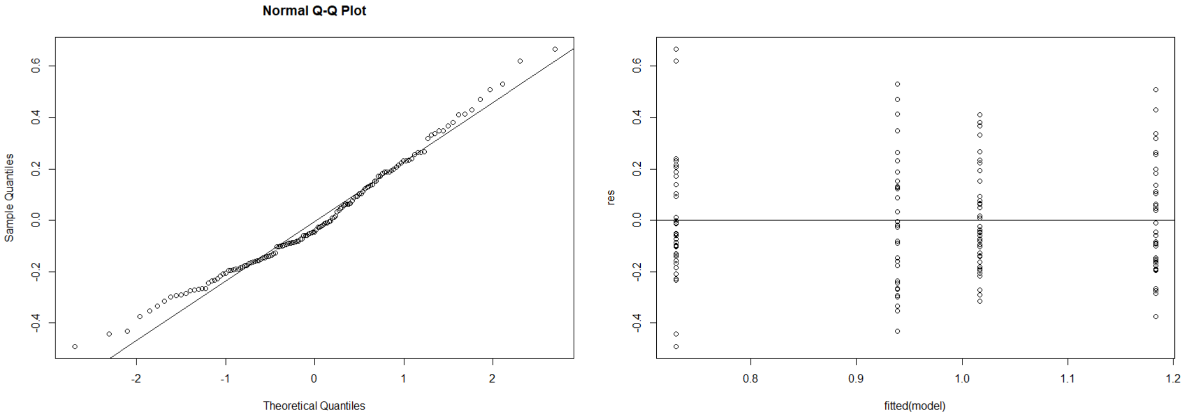

Fig. S4 Q-Q plot and residual vs fitted plot of total PME activity in pollinated silks in the *Ga2* study

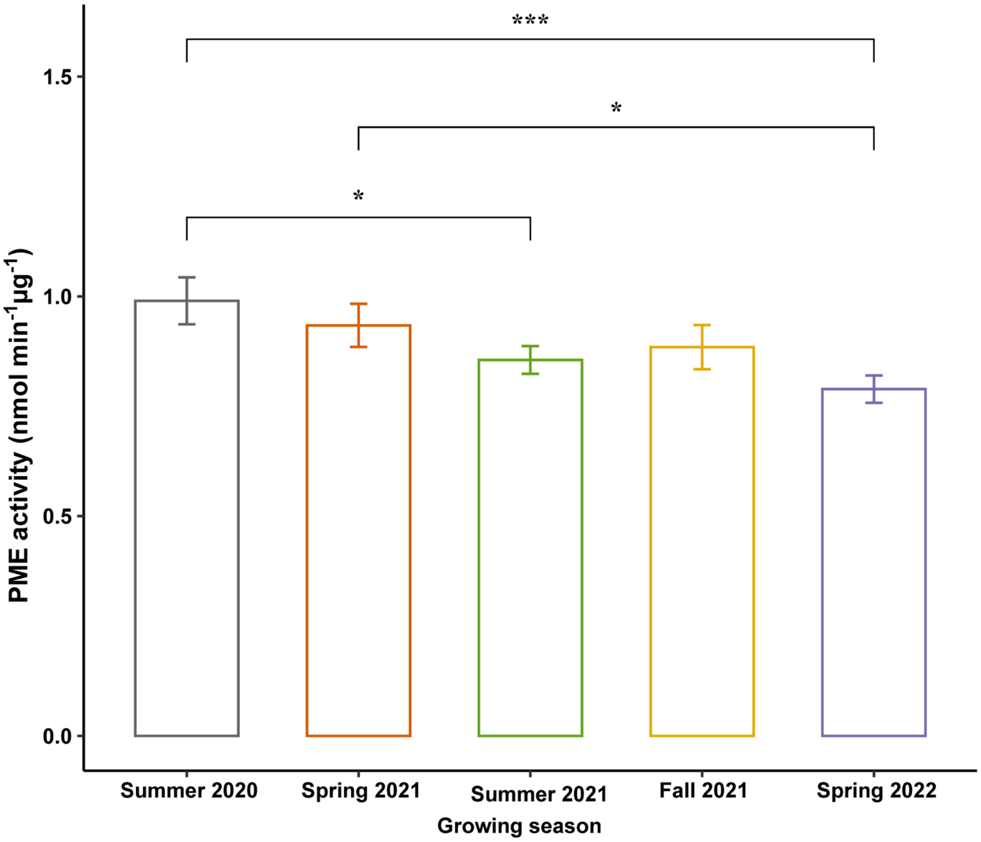

Fig. S5 Growing season-wise differences in total PME activities. Bar plot represents the means ± SE of the groups.

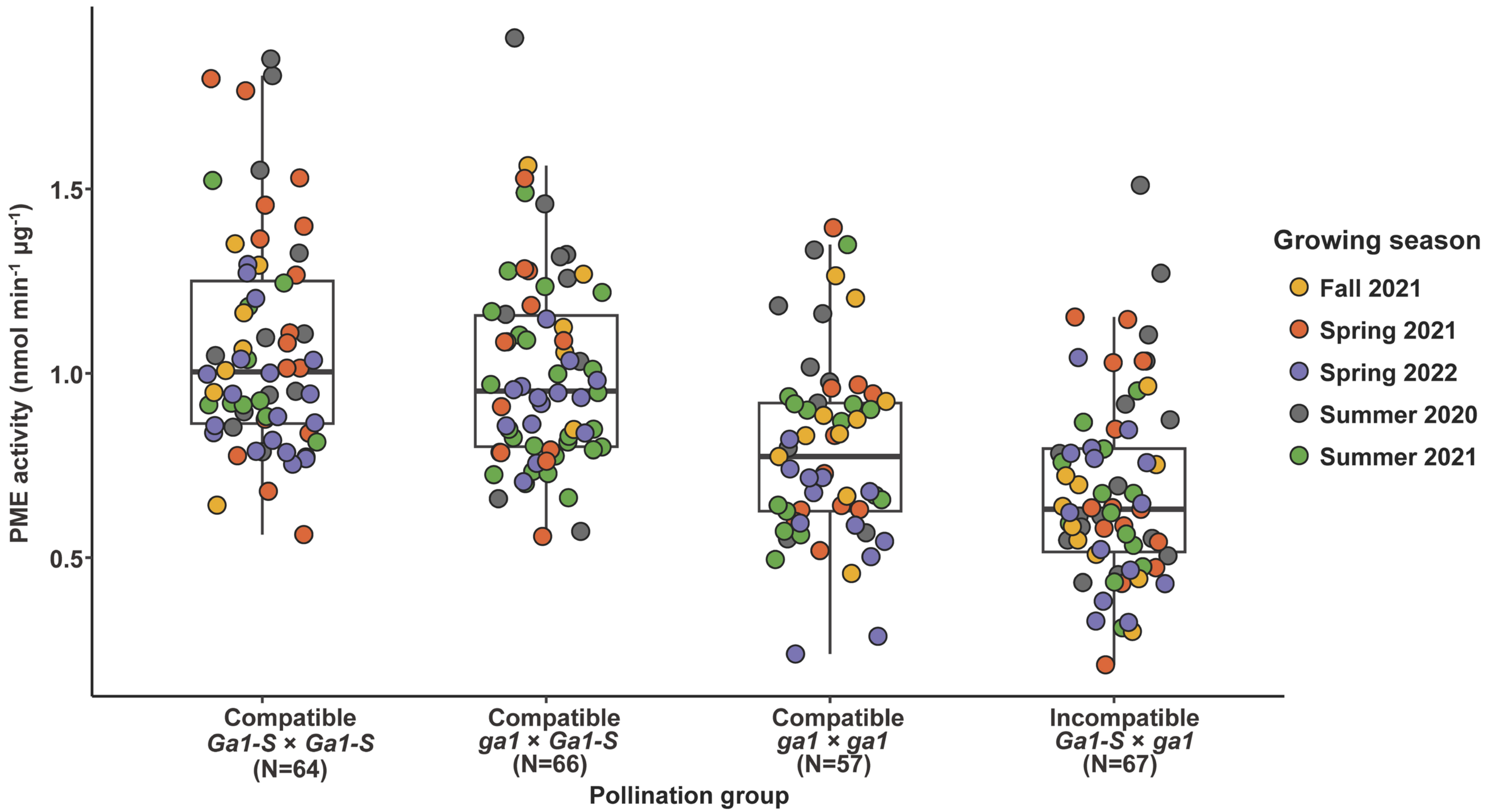

Fig. S6 Total PME activity in compatible and incompatible crosses involving *Ga1-S* and *ga1* haplotypes across 5 growing seasons

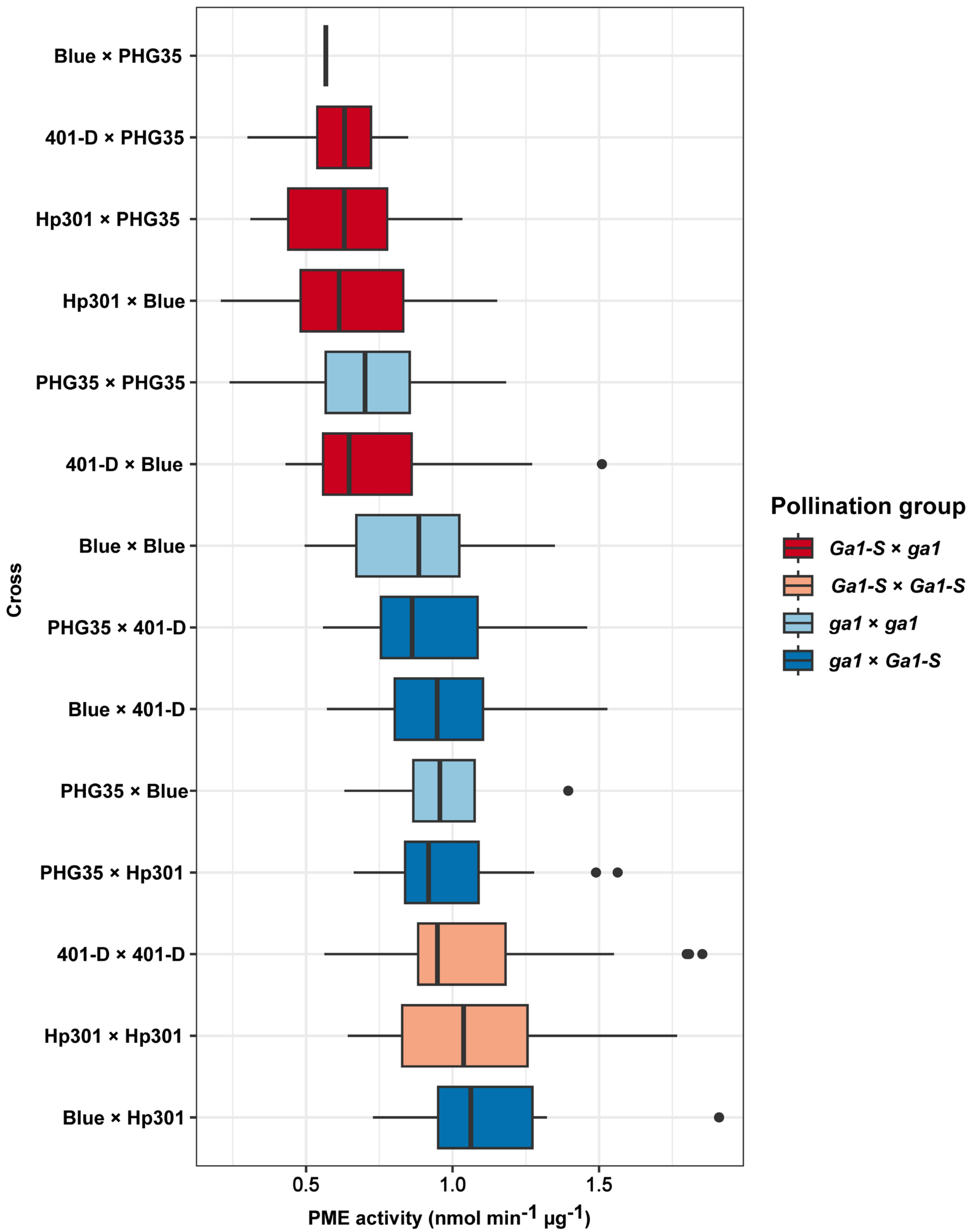

Fig. S7 Crosswise total PME activities in the *Ga1* study

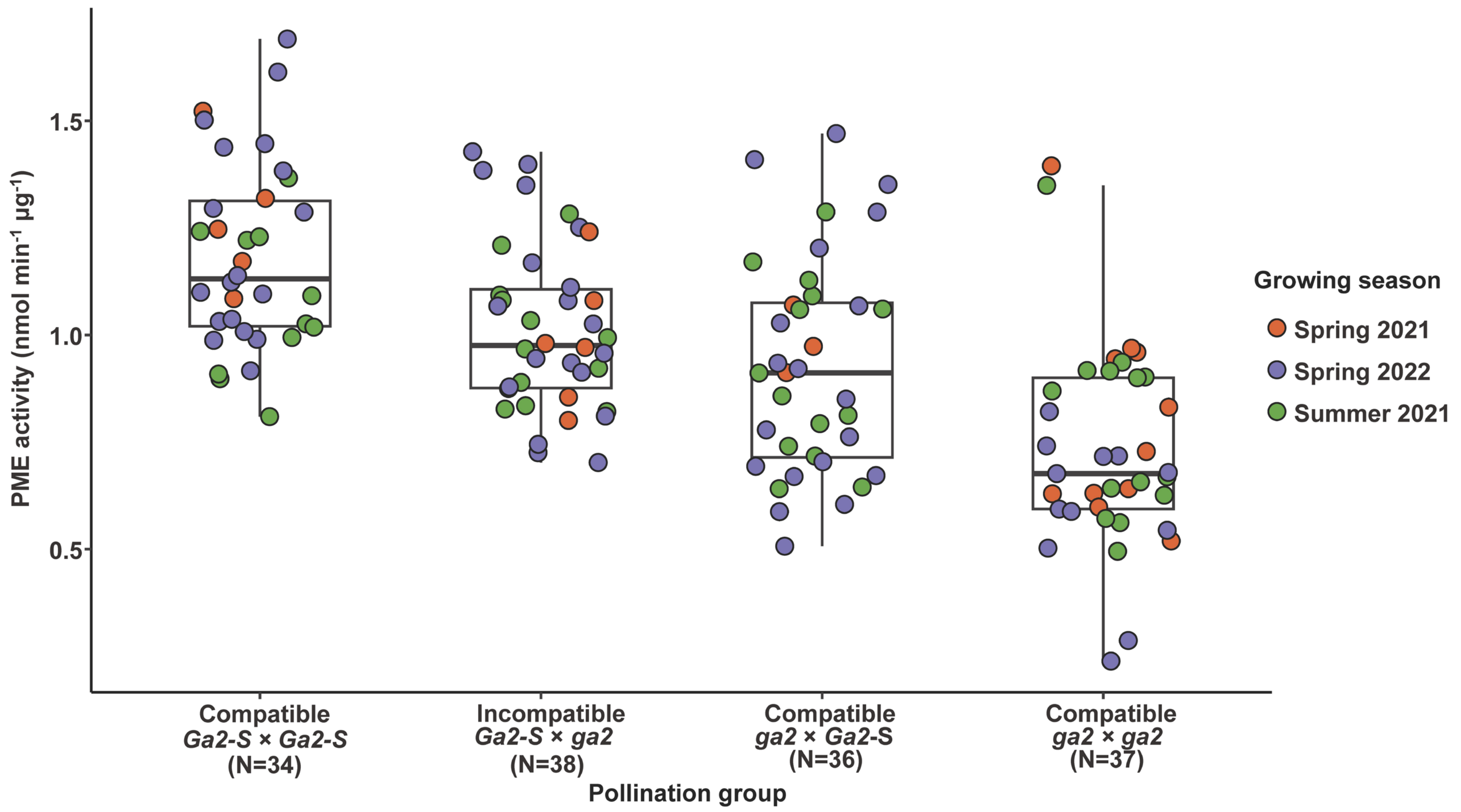

Fig. S8 Total PME activity in compatible and incompatible crosses involving *Ga2-S* and *ga2* genotypes across three growing seasons.

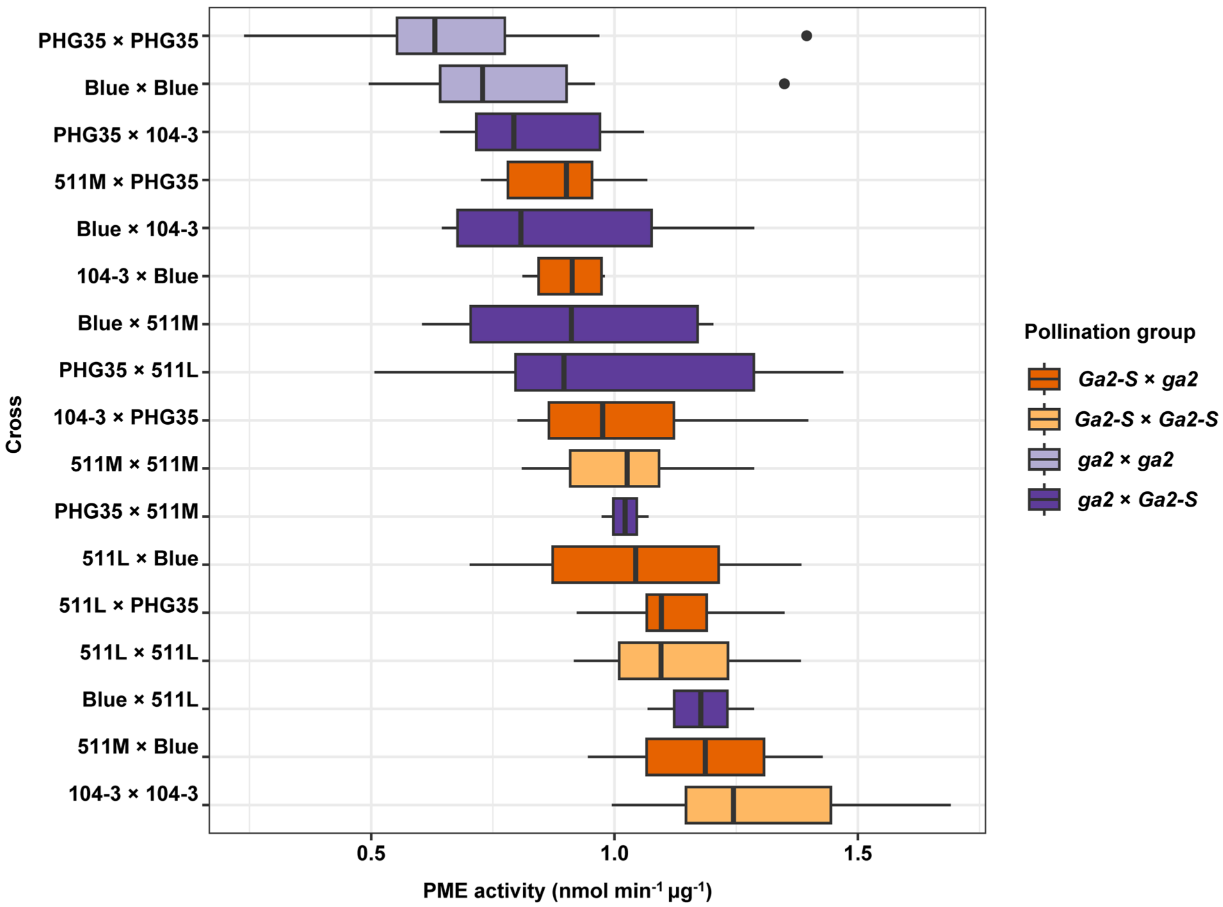

Fig. S9 Crosswise PME activity across different pollination groups belonging to *Ga2* cross incompatibility

```
D_carota            -----KHQAVALRVGSDLSAFYRCDILAYQDSLYVHSNRQFFI-NCFIAGTVDFIFGNAA   164
ZmGa2F              -----GRQAVALRSNSNRTVVFGCAIEGFEDSLYAENGVQVYL-ETDIYGTVDFIFGNAK   207
Tcb1-f              -----GRQAVALRSNSNKSVVYWCSIEGHEDTLYVENGIQFYL-QTSIWGTVDFVFGNAQ   234
ZmPME3              -----GRQAVALRSNSNKSVVYCCSIEGHEDTLYVENGIQFYL-QTSIWGTVDFVFGNAQ   234
ZmGa2P              KKKKAEGEAPALRVLGTKATFYNCTIEGGQGALYDQMGLHYFK-SCTIRGTIDFIFGSAK   254
Tcb1-m              KPGEKKGEAPALRVMGTKATFYNCTIEGGQGALYDQTGLHYFK-ACAIKGTIDFIFGSAK   239
ZmGa1P              KLGEKKGEAPALRVMGTKATFYNCTIEGGQGALYDQTGLHYFK-ACAIKGTIDFIFGSAK   239
ACF79840.1          ----DNGPAVAAKVDADKAAFYDCRFLSYQDTLLDATGRHYYR-GCYIEGATDFIFGTGK   197
AQK71914.1          EEGVLALLELKVRVEADPHGIQDWDPADAAGLAYGALTLTFVIIDEWDVNSICRRFTLHT   211
NP_001144148.1      KPGAKGGQAVAVRLFGTKTQIYNCTIDGGQDTLYDHKGLHYFK-GCLIRGSVDFIFGFGR   247
NP_001146436.1      LVNDPEIRSVAAMVAGDKVAFYHCAFYSPHHTLFDSAGRHYYE-SCYIQGNIDFIFGSGQ   209
NP_001151351.1      ----GSGQAVAVRVTADRCAFYNCRFLGWQDTLYLHYGKQYLR-DCYIEGHCDFIFGNSV   179
NP_001167796.1      KPGAAGKQAVALRVSADNAAFVGCRFLGAQDTLYDHSGRHYYK-DCYIQGSVDFIFGNAL   268
NP_001296949.1      MPGTQGGQAVAFRISGDKAFFFGCGFYGAQDTLCDDAGRHYFR-DCYIEGSIDFVFGNAR   215
XP_008649911.1      RPGALGKQGVALRISADSAAFVGCNFLGAQDTLYDHLGRHYYR-DCYIEGSVDFIFGNAL   279
XP_008653334.1      PPGAVGQQAVALRLSGDKTMLYRCRILGTQDTLFDNIGRHFLY-NCDIQGSIDFIFGNAR   235
XP_008653773.2      DPGDAGGQAVALRVRGDQAAFYWCGFYSSQDTLLDEQGRHFFR-GCYVEGSIDFIFGNAR   255
XP_008655860.1      G---RIAPAVAALAAGDRSSFYRCGFVSVQDTLSDLEGRHYYE-GCYIEGAMDFIFGNGQ   201
XP_008656474.2      RPGALGKQGVALRISADNAAFVGCNFLGAQDTLYDHLGRHYYR-DCYIEGSVDFIFGNAL   266
XP_008657240.1      RPGATGKQAVALRVSADNAAFVGCSFLGAQDTLYDHSGRHYYK-ECYIQGSVDFIFGNAL   264
XP_008660472.1      APGQEGGQAVALRVFGTKAAFYDCTIEGGQDTLYDHKGLHYFK-SCHIQGSVDFIFGFGR   264
XP_008662740.1      RPGAVDAQAVAIRINGDQAAFWGCGFFGAQDTLHDDRGRHYFK-ECFIQGSIDFIFGDAR   279
XP_020398192.1      -----SGPAVAVRVAGDRAAFYGCRFTSFQDTLLDDTGRHYYR-GCYVQGGTDFVFGNGK   124
                                   .    .                            .     *

D_carota            PVQ---SSFPTYLGRPWKEYSRTVVMQSSITNVINPAGWFPWDGNFAL----DTLYYGEY   266
ZmGa2F              PVGQNLTGVETFLGRPYRKYSHVIFMECQLSDVVSAAGWVAWDRAHVINDTTKSVRYMEY   317
Tcb1-f              --GVDLDGVETYLGRPYRNFSHVAFIKSYLSRVVSPNGWVAWNKNKVVEDTTRTILYLEY   338
ZmPME3              --GVDLDGVETYLGRPYRNFSHVAFIKSYLSRVVSPNGWVAWNKNKVVDDTTRTILYLEY   338
ZmGa2P              --------QQIFLGRMGT---PSIYSYTQIAKEVVPIIYDKGNIF-M-PSNMTGRRCATF   355
Tcb1-m              --------EKIYLGRVGT---PVIYSYTNIGKEIVGIISNGQDV----QTVERGYYCATF   336
ZmGa1P              --------EKIYLGRVGT---PVIYSYTNIGKEIVGIISDGRDV----QTVERGYYCATF   336
ACF79840.1          -------VATAILGRPWGPYARVVFALCNMSNTVAPEGWNNWDNTA----NEKTAFFGQF   289
AQK71914.1          PLVAN---ISSYLGRPWKRYSRAVFAQTKMEALVHPRGWLEWNATF----ALDTLYYAEY   316
NP_001144148.1      -------GGQIYLGRAWGDSSRVVYSYTKMGEEVVPVGWDGWQIAK---PESSGIYYGEF   343
NP_001146436.1      ---------EVYLGRVTAPDSRVIFADTYLSKTIHPAGWTTIGYS----GSTDKVTLAEF   302
NP_001151351.1      ------EAGYMFLGRPWGPFGRVVFAYTFMDRCIKPSGWHNWDKS----ENERTACFYEY   270
NP_001167796.1      ---------ALYLGRAWGTFSRVVFAYTHMDDIIVPNGWFNWGDP----NRELTVFYGQY   358
NP_001296949.1      ---------RLYVGRAMGQYSRIVYAYTYFDSVIAPGGWDDWDHTS---NKSMTAFFGMY   306
XP_008649911.1      ---------ALYLGRAWGTFSRVVFAYTYMDNIIIPRGWYNWGDP----TREMTVFYGQY   369
XP_008653334.1      ---------MLYLGRAWGRYARVVYSYCDLGGIVVPQGWSDWGDQ----SRTKTVLFGEY   325
XP_008653773.2      ---------QVWLGRAWGPYATVVFARTYLSAVVAPAGWNDWNDP----ARQQSVFFGEY   351
XP_008655860.1      ---------PAYLGRAWRRYARVIFFQTDMSGVVVSQGWDAWSYK----GTEGTLTMVEE   293
XP_008656474.2      ---------ALYLGRAWGTFSRVVFAYTYMDNIIIPRGWYNWGDP----TREMTVFYGQY   356
XP_008657240.1      ---------ALYLGRAWGTFSRVVFAYTYMDDIIIPKGWYNWGDP----NRELTVFYGQY   354
XP_008660472.1      -------AGQIYLGRAWGDSSRVVYSYTTMGKEVVPVGWDGWRIER---PEKSGIYYGEY   360
XP_008662740.1      ---------SIWLGRAWRPYSRVIFAYTSMSDIIASEGWNDWNDQ----TRDQTVFYGEY   377
XP_020398192.1      -------AGTSVLGRPWGPYSRVVFALSYMSGTVRPQGWDDWSDSSR-QSRSRTAFYGQY   219
                                :**              :   :
```

Fig. S10 Partial multiple sequence alignment of D. carota PME, *Ga1*, *Ga2* and *Tcb1* male and female function PME determinants and Type II PMEs in the maize PME family. Conserved active site residues are marked in blue text and active site mutations are marked in red text
